# Consumers’ Loyalty Related to Labor Inclusion of People with Disabilities

**DOI:** 10.3389/fpsyg.2016.00885

**Published:** 2016-06-21

**Authors:** Marta González, José Luis Fernández

**Affiliations:** Chair in Economic and Business Ethics, Comillas Pontifical University, MadridSpain

**Keywords:** responsible consumer, loyalty, disability, functional diversity, labor inclusion, certifications

## Abstract

**Purpose:** the purpose of this paper is to show that reporting the corporate commitment to labor exclusion of people with disability correlates with the increase of consumer loyalty.

**Methodology:** It is a theoretical revision that will relate consumer loyalty to three main topics: disability and labor exclusion, responsible consumerism toward disability, and corporate communication to increase loyalty of those consumers that are concerned about this problem.

Findings:

• Disability is an invisible phenomenon that concerns the whole of human society. So, the exclusion of the collective appears as a great social problem that might be dealt by the companies to be perceived as responsible.

• Responsible companies are awarded with the loyalty of the consumers.

• Clear corporate information about the commitment with this problem will reinforce the loyalty toward the brand.

• This information can be given in an informal way or by following a certification process. The impact of those methods will depend on how disability is understood by each consumer.

**Originality/value:** This paper focuses on a topic usually neglected by companies and even by literature. However, the fact that more and more companies are paying attention to this problem allows us to think that we are facing a social change that will challenge companies.

## Introduction

This paper focuses on how companies inform society about the commitment they hold with the labor exclusion of people with disabilities. We sustain the hypothesis that this information will increase the loyalty of those responsible consumers who take upon themselves to check if the products they acquire take into account the hopes and wishes of this group of people regarding their labor inclusion.

The methodology that we will use to prove the consistence of our statement will be a theoretical revision. It will link the concept of consumer loyalty to three topics: disability and labor exclusion, responsible consumerism toward disability, and the forms used by the companies to increase loyalty of those consumers that are concerned about this problem. Throughout the literature it will be shown that more and more companies are reporting about their commitment with this problem, even the exclusion suffered by the collective is not widely known. It seems that they are sending a message for those consumers that could be interested in this challenge. Different methods of doing it will be exposed. As a line of research, the kind of information and how to provide it to satisfy this social concern remains to be determined.

This topic is important considering that 15% of population has a disability (WHO, 2011) and that this percentage will increase due to chronic diseases that will be overcome due to the increasing age of the population (Angeloni, 2013). On the other hand, the Council for Disability Awareness (CDA, 2013) highlights that 25% of young people that are 20 years old will suffer a disability before finishing their professional career. Moreover, we can’t forget that behind a person with a disability there is almost always a family who directly or indirectly suffer or share the hardships of their relative with a disability (Angeloni, 2013; Adecco, 2014).

We can also say that we are facing a phenomenon that deserves attention for demographic reasons. Thus, it goes further than a marginal character of disability, but it concerns everyone. It may seem like an uncomfortable truth, but sooner or later everybody, will face some type of disability. Despite the fact that it is not widely known, nor envisioned in the long term (Coe and Belbase, 2015), physical and mental vulnerability are conditions that everyone experiences in the course of their own existence (Reynolds, 2008). With this in mind, the way that companies inform society about how they respond to the concerns about disability becomes crucial when talking about loyalty of potential costumers (Valor et al., 2012).

The concept of customer loyalty has received considerable attention in the marketing literature. In this sense, several researchers explain loyalty purely from the behavioral point of view (Jaiswal and Niraj, 2011), whilst some argue that an attitudinal perspective is more reflective of customer loyalty (Jacoby and Chestnut, 1978; Flint et al., 2011). Regarding the loyalty to a brand related to the labor inclusion of people with disabilities, our point of view embraces an integrated theory which suggests that customer loyalty is a combination of both behavioral and attitudinal loyalty (Dick and Basu, 1994; Oliver, 1999). This way of thinking is consistent with the main goal of this paper, which aims to link labor inclusion of people with disabilities, responsible consumerism, and loyalty to a brand.

Limited studies examine consumer’s attitudes toward hiring people with disabilities. However, a national survey in USA found that 92% of consumers felt more favorable toward companies that hired them because it was assumed that these firms would care about their workers. Another positive aspect that was pointed out was that around 83% of the participants felt that companies did not take advantage of their workers with disabilities, nor did people with disabilities create problems in the workforce. Almost all of those surveyed (96%) shared the belief that companies who hire people with disabilities help those individuals to lead more productive lives. Furthermore, by including people with disabilities in their workforce, the participants also viewed companies that helped their employees as having a better understanding of people with disabilities. Finally, over one third of the survey group agreed that they would prefer to give their business to companies that hire people with disabilities (Siperstein et al., 2006). The results of this study show that the public view of hiring a person with a disability is considered as the socially responsible thing to do, as well as a gainful business practice. Thus, given these data, companies would have to find the right way to communicate this practice to the consumer because studies suggest that when the actions held by the companies are perceived as socially responsible they will influence costumer’s purchase intention (Ajzen and Fishbein, 1980; Kuo and Kalargyrou, 2014).

Even the good perspectives and results given by the findings shown by the findings above, the labor exclusion of people with disabilities appears to continue over time. We can point out that in Spain a survey conducted by the NSI from 2009 to 2012 (NSI, 2013) showed that the employment of people with disabilities decreased gradually over that period, from 28.3 to 24.5%, in men, and from 23.7 to 22.5% in women. Unemployment, on the other hand, rose to a large extent, exceeding 6.2% in that of the population without disabilities. Unfortunately, this situation seems to be similar everywhere in Europe (ESS, 2010).

Several studies suggest that disability is one of the main causes of poverty all over the world (Laparra et al., 2007; Loeb et al., 2008; Braithwaite and Mont, 2009; Mitra et al., 2011; Martínez, 2013). This is due not only to the objective limitations of their state of health, but also to the fact that they tend to hold the worst positions when they can find a job (Malo and Muñoz-Bullón, 2006; Jiménez-Lara and Huete García, 2011). Additionally, they usually have a lesser income than the average population (Humer et al., 2007; Jones et al., 2007; Brown and Emery, 2008), and must meet the additional costs that their disability entails: care workers, removal of architectural obstacles in their homes, and medicines, among others (Durán, 2002; Parckar, 2008). For that matter, the arrival of disability in the home involves personal costs, particularly for women (García et al., 2004; Torns and Recio, 2012), who on many occasions are forced to give up their careers to look after the person directly affected by the problem (Malo, 2004; CERMI, 2012; Martínez, 2013; Adecco, 2014).

The controversial point of this paper is that despite the high percentage of population affected by some type of disability, the labor exclusion experienced by them, and its bad consequences, is still unknown by the large part of society and companies. It is widely accepted that economic help and assistance is the best solution to the problem (Peloza and Shang, 2011; Browne and Nuttal, 2013; González, 2015). Even so, we insist on the importance of communicating to society, in a measurable and assessable way, that labor inclusion of people with disabilities is a main challenge for the company.

Nevertheless, neglecting their labor inclusion has for years been legitimized by citizens who understood the labor exclusion of people with disabilities as a part of their condition of being “incapable” to work (Berger and Luckmann, 1971; Albrecht and Levy, 1981; Bickenbach, 1993); even the collective itself understood it, given their limitations to carry out “normal” activity (Seligman, 1972; Jost et al., 2003).

It is true that the concept of disability has changed over the years. It has been linked to several paradigms that had considered it as a problem to be eradicated to accepting it as a part of society that needs to be healed and supported to become “normal” (Wolfensberger, 1975; Casado, 1991; Aguado, 1993). Under this way of thinking people with disabilities were accepted in the workplace, but their performance had to be equivalent to the other workers in the company (González, 2015). It appears, however, that way of understanding reality is starting to change, and more people are increasingly demanding companies to adapt the working environment to guarantee that people with disabilities can reach the labor market in equal conditions as others (Asís and Barranco, 2010).

We point out that this claim is made under a new paradigm of disability, in which the dignity of people and their moral autonomy are underlined. From this approach, disability is to be called “functional diversity”, describing a vital situation in which people function in a different way to the majority of others (Romañach and Palacios, 2008; Ferreira, 2010). In this sense, the negative medical connotations that have historically accompanied this collective, under the clinical model, have disapeared (Romañach and Lobato, 2005; Palacios and Romañach, 2006).

This new way of facing disability was reflected in the International Convention on the Rights of People with Disabilities (2006), which focuses on equal opportunities, equal rights, and the dignity of people. The main effect of this conceptual change has been to consider those with disabilities as people with rights and not objects of charitable policies. It also entails understanding that the social disadvantage is a clear example of discrimination and breaches of human rights (Asís and Barranco, 2010).

However, as the employment results show, it appears that companies are not reflecting this new discourse. Companies, in general, are attempting to help people with disabilities with cause-related marketing and philanthropy actions (Min-Young et al., 2009; Peloza and Shang, 2011; Vauclair and Fischer, 2011). These kinds of actions are usually seen by society as a good thing (González, 2015), and therefore they are legitimized (Berger and Luckmann, 1971). But, if we bear in mind the new demands of the collective, it seems clear that the answer put forward by “benevolent companies” (Zamagni, 2012) is insufficient. Going further on this topic, we would dare to say that this way of marketing could be considered as harmful for society at large, because it leads to harmful consumption decisions (Smith et al., 2010). In other words, whenever a consumption behavior is influenced by this kind of publicity, the consumer is accepting benevolence and is neglecting the possibility of including people with disabilities into the value chain of the company.

Considering what we have exposed above, labor exclusion is a significant problem that is gradually coming to light and one which companies should respond to if they want to speak of responsible management (Freeman and Reed, 1983; Freeman, 1984; Carroll, 1991; Mitchell et al., 1997; Freeman and Philips, 2002; Young, 2003; Freeman et al., 2007).

Thus, there is a new character among the consumers that will pay special attention to the way the companies deal with this problem. In this paper we call this character a “consumer socially responsible toward disability”, and we will link his behavior with Corporate Social Responsibility. In this sense, we consider responsible management as a preceding that includes, in a strategic way, the expectations and concerns of those persons that are around the company and are affected by it (Freeman, 1984, 2012; Carroll, 1999).

To be perceived as responsible toward the inclusion of people with disabilities, companies develop different actions to show society their policies in favor of these people. Some organizations show this through social events where their task is recognized and others show their commitment with a seal obtained by a certification process. In this paper we will focus on two kinds of certificates that are currently used to report business activity in the area of disability. The first one is already extended in more than thirteen countries and would label the firm as “Certified Professional in Disability Management” (CPDM). They work following the pattern established by the Integrated Disability Management (IDM). Its main goal is to avoid that disability blocks the professional career because of its arrival. The second one has been present in Spain since 2013. It is called the Bequal certificate and it analyzes the inclusion of disability following the criteria established by the Convention on the Rights of People with Disabilities (UNO, 2006). It can be said that these two certificates are quite different in their philosophy and about what they want to measure, but both of them gather most of the concerns of society regarding disability. These three models are not exclusive, as they could be used in a complementary way.

## Socially Responsible Consumers Toward Disability

Socially responsible consumers (SRCs) are defined as citizens who are not just interested in satisfying their own needs, but who integrate their concerns for the environment and social causes into their purchase decisions (Arredondo et al., 2011), in a regular way (Stolle and Hooghe, 2004), and as a part of their personal project (Little, 1993). This kind of purchasing constitutes and expresses their identity (Newholm and Shaw, 2007), and their response to social or environmental issues does not come exclusively from cause-related marketing campaigns launched by companies (Roberts, 1996; Carrington et al., 2010; Smith et al., 2010). Consequently, a SRCs can be defined as the one that considers his acts of consumption as a chance to preserve the environment and the quality of life in a local context. In this sense, the SRC refuse those products that are dangerous for health, those that are packed in a non-ecological way, or might be harmful because of the material employed to produce it. At the same time, this consumer takes into consideration the responsible behavior of the companies and those products offered by fair trade. It can be said that price is not the only factor considered when the product is being purchased anymore (Akehurst et al., 2012).

It has been proved that the information given by the company, the Corporate Social Responsibility strategy, and the purchase intention are all related (Lee and Shin, 2010). The positive influence of Corporate Social Responsibility on consumer behavior has been shown by literature even when the price of the product increase (Mohr and Webb, 2005; Alvarado and Schlesinger, 2008; National Geographic and GlobeScan, 2012). For this to be so, there is a relevant condition: the more knowledge and trust the consumer has about Corporate Social Responsibility, the better the response is (Tian et al., 2011; Kozar and Hiller Connell, 2013). In addition, if the beneficiaries of this socially responsible product are people, instead of animals or environment, the willingness to pay for it will increase (Tully and Winer, 2014). Something that has been pointed out is that women are more susceptible to support Corporate Social Responsibility than men (Arredondo et al., 2011) and are more likely to be influenced in their purchasing decision after a marketing related campaign (Hyllegard et al., 2010). But be that as it may, quality of the product is the main aspect considered by all consumers, including those who are called socially responsible (Feldman and Reficco, 2015).

In any case, the process to become a responsible consumer seems to follow a pattern strongly influenced by life experience. The process of becoming a responsible consumer is fostered during the person’s childhood due to family values (Valor et al., 2012). The social environment will help to build their personal project. In addition to that mentioned so far, the degree of consumer knowledge of the problem they are trying to alleviate, and the perception of self-efficacy when engaging in a responsible purchase has to be considered (Gupta and Ogden, 2009; Valor et al., 2012). It is also important to bear in mind age, gender (Arredondo et al., 2011), socio-economic status (Fraj and Martínez, 2003), pertaining to a group involved in the defense or protection of social causes, and personal values (Fraj et al., 2004; Valor, 2008; Gupta and Ogden, 2009; Valor et al., 2012).

It appears that the character of responsible consumers has acquired great importance in recent years, and, as we anticipated, companies have to adjust to these new demands and find a way of making society aware of it if they truly want to influence the behavior of consumers (Camacho et al., 2013) and be sustainable (Moneva and Ortás, 2010; Fraile and Fradejas, 2012; Orozco and Ferré, 2013; Retolaza et al., 2014).

Not only do these new consumers question the way companies work, but they can also boycott their products or services (Paeck and Nelson, 2009) if they believe that they are not the fruits of responsible management (Freeman, 1984; McAlister and Ferrell, 2002; Porter and Kramer, 2006). Consequently, current companies must be prepared to offer information that differentiates them from their competitors. Such information must express not only the quality of the product or service, but also the values of the company and its management of its chain of worth (Auger et al., 2008; Russell and Russell, 2010; Shandwick, 2011).

Nevertheless, to be a responsible consumer is not an easy task. There are obvious barriers against putting the intention of responsible purchasing into practice, namely, the lack of time of consumers or the difference in price regarding other products (Beckmann, 2007), the difficulty in finding appropriate information on the chain of worth of companies that offer the service, and the lack of these types of products in normal consumer points of purchase (Valor, 2008; Carrington et al., 2010; Arredondo et al., 2011).

In line with, among other things, responding to this type of consumer, Corporate Social Responsibility has been increasingly emerging (Garriga and Melé, 2004; Fernández, 2007). This business model will seek to include social concerns into its management method (Freeman, 1984), without neglecting financial profit (Freeman, 1984; Kelly and White, 2009; Canals, 2010; Krauss and Brtitzelmaier, 2012).

Some institutions have developed verifiable management models, of voluntary application, to facilitate dialog with different affected public groups and to make society aware of the management of companies. Significant examples of these verifications are the Global Reporting Initiative (GRI) guidelines, the AA1000 model, the SA8000, the SGE21 standards, and the IS0 26000 recommendations and guidelines. Some of those are even certifiable through independent agencies, and, although all are voluntary, provide useful information when interpreting and comparing management quality and the companies performance (Andreu and Fernández-Fernández, 2011). It is noteworthy to underline that implementing socially responsible policies generate client loyalty, differentiates them from the competition, and attracts talent, investment and “responsible” consumers, and improves the working environment (Bhattacharya and Sen, 2004; Bhattacharya et al., 2009).

However, these tools have overlooked the existence in companies of people with disabilities. Evidence of this is that none of the standardized instruments for business auditing in the responsible management area has specific indicators on the inclusion of disabled people in companies. It is true that sustainability reports include sections on the Corporate Social Action (Moneva and Ortás, 2010), in which diverse activities such as sponsorship or voluntary actions in favor of people with disabilities are described. In this sense, nothing registers markers to measure how equal access to employment is guaranteed (Asís et al., 2007). In fact, it was not until 2013 that these parameters were included (GRI-ONCE, 2013). The involvement of people with disabilities in adding parameters that could report objectively the commitment of the companies can be considered as an indicator that they don’t want help or charity but work.

In order to give a response to this new social demand, in the next section we present two main approaches to certify that disability is being taken into account within the companies.

## Reporting Corporate Commitment With Disability to Improve Consumer Loyalty

As we have already explained, the loyalty of the costumers comes from considering that the company is running the business in a responsible way. In other words, the costumer will be prone to maintain a purchasing pattern whenever he/she will feel that the firm has a behavior connected with his/her concerns. In this sense, companies will have to make a special effort to let society know how management is.

As Oliver proposed (1999), this information given by the company will mean the first step to build loyalty. This author defined a pattern that starts by the *cognitive* comprehension of the information given by the brand. If it is accepted by the consumer, and brings a pleasurable fulfillment, it will become an *affective loyalty* showed in a positive attitude toward the brand. It goes without saying that if people perceive and understand that the brand is taking their needs and concerns into consideration, they will have a positive attitude toward the organization and its products and services. The next phase of loyalty development is the *conative* stage, where the consumer feels committed to purchase.

Following this idea, for some time now, several companies look for the acknowledgment of society of the role they are playing toward the exclusion of people with disabilities (Valor, 2004). To get it, they hold parties and events where they can assure their good practices are visible. Prizes and awards are given receiving media support. The main idea is to be perceived as an ethical brand, as a company that doesn’t harm, but instead promotes it showing its honesty, integrity, diversity, responsibility, quality, respect, and accountability (Fan, 2005).

Despite being an obvious method of showing the reality of people with disabilities and a useful way of demonstrate to society that ways to eradicate exclusion exist, there is no doubt that the evaluation carried out of the proposals is neither official, nor has institutional recognition, let alone a standardized report, or control procedure. The visibility and impact of these types of actions appears somewhat limited, and, as it has been said, harmful for people with disabilities.

In other words, analyzing the value chain is needed to get to know how deep the commitment is that the company holds with people with disabilities and to endorse it.

To give verifiable information about how the company is managing diversity, and specifically disability, companies are looking to be certified. These certifications can focus on different aspects. As it has already been said, the CPDM seal will take into consideration how managers deal with disability in the company to avoid or decrease the risks and consequences of a disease that could turn into disability. It is reasonable to think that this approach will be accepted by a large group of population that considers that disability as a problem that has to be eliminated or healed to become “normal”. But as it has been explained, the new claims of people with functional diversity consider that what causes the disability are the barriers that the person finds to be included. In this sense, the Bequal Certificate could generate more loyalty among the citizens that are trying to make society understand that diversity has to be respected.

Thus, the designation of “Certified Professional in Disability Management” (CPDM), offered by IEA (Insurance Educational Association) and DMEC^1^ is given to companies that can demonstrate knowledge on the following areas: disability and work interruption case management; workplace intervention for disability prevention; program development, management, and evaluation; and employment leaves and benefits administration^2^. The CPDM designation is an internationally recognized certification accredited by the International Disability Management Standards Council (IDMSC) within 13 countries, namely, Australia, Austria, Belgium, France, Luxembourg, the Netherlands, Canada, Germany, Hong Kong, Ireland, New Zealand, Switzerland, and UK^3^. It can be used as a seal to improve communication and marketing to win new customers, new suppliers, and new partners. In fact, the growing public attention to the issues accentuates the level of ethical maturity of all stakeholders, which will reward companies that invest in health and safety (Angeloni, 2013).

In this sense the management of the company will follow an IDM that will pay special attention to any kind of disability that might overcome the workers of the company: specific health risks (e.g., physical inactivity, poor nutrition, tobacco use, stress, depression), conditions (e.g., obesity, muscle-skeletal disorders, mental health), and diseases (e.g., heart disease and stroke, high blood pressure, diabetes, high cholesterol, cancer, arthritis) can be addressed (Rieth et al., 1995; Calkins et al., 2000; Shrey et al., 2006; Rosenthal et al., 2007; Habeck et al., 2010; Angeloni, 2013).

In turn, the Bequal certificate would recognize those companies where managers could demonstrate that policies to avoid discrimination and that guarantee equal opportunities across all the areas of the are being implemented. Same criteria should be followed for suppliers, and has to be included in the implementation of occupational risk prevention. Special attention has to be paid to the existing limits for people with a different way of functioning: mobility barriers, communication and information barriers, or access to the product or service given by the company, and negative attitudes (Asís et al., 2007). If this analysis is not carried out, the information taken about how the company includes disability may overlook the heterogeneity of people with disabilities (Díaz-Velázquez, 2010; Ferreira, 2010).

It is worthy to mention that the Bequal certificate has been created by the most important organizations that represent people with disabilities in Spain^4^. It can be said that with this certificate they are giving voice and solution to most of their concerns, according to their personal experience (Valor et al., 2012), and that they are proposing an alternative themselves. Their participation in those decisions that affect their quality of life becomes vital to be supported by consumers (Stanaland et al., 2011).

Even though its implementation and actual scope can seem to be short-sighted, it is being implementing not only in national companies, but in several multinational companies such as: Repsol, Acciona or Gas Natural Fenosa, all of them having strong programs of Corporate Social Responsibility.

The certification granted by Bequal focuses on aspects that have not been considered by others standards to date:

(1) It looks at the strategy and leadership of the company.
(2) It commits to people with disabilities.
(3) It looks at the management of Human Resources as it has been established by Law on the Social Integration of Disabled Persons [LISMI] (1982) regarding: recruitment, selection, promotion and other tasks inherent to the position.
(4) It analyzes if a homogeneous distribution of disability exists.
(5) It certifies responsible purchasing.
(6) It evaluates the accessibility of products and the attention paid to clients with disabilities.
(7) It evaluates the social action developed by the company.
(8) It looks at the style of the internal and external communication of the company.
(9) It observes the language used to transmit the information of the company in equal conditions.

Thanks to these seals, companies can give reliable data that will offer understandable information about their way of running the business and their relationship with people with disabilities. In short, both certificates will report to the company *cognitive and pragmatic legitimacy* (Suchman, 1995). In this way, society will understand how management is held, how and why things are done. It will facilitate that the purchasing intention becomes a real fact in a sustainable way (Fombrun, 1996; Deephouse, 2000; Basdeo et al., 2006; Helm, 2007; Schwaiger et al., 2009). The more information the company facilitates, the more reliable it will be for the community (Berger and Luckmann, 1971; Suchman, 1995; Carreras et al., 2013).

Advantages to the company that will be rewarded by these seals will come from the fact of using a tool where the main point is to give an answer to expectations and concerns of people with a disability, or with a potential disability, and their families. In this sense, the behavior of the company is perceived as authentic and aligned with the values of people that could be affected by this problem (Becker-Olsen et al., 2006; Varman and Belk, 2009; Wagner et al., 2009; Ruiz de Maya et al., 2015).

As a result of this genuine collaboration, literature in marketing points out the following benefits for the company: greater sales volume, enhanced operating efficiencies, positive word of mouth, reduced marketing expenses, and enhanced consumer loyalty (Reichheld and Sasser, 1990). In addition, it has been found that consumer participation has a positive effect on consumer behavior (Dellande et al., 2004; Chan et al., 2010) which turns into brand loyalty (Bagozzi and Dholakia, 2006), commitment to the brand (Casaló et al., 2007), quality perceptions (Dabholkar, 1996), trust (Ouschan et al., 2006), and affective commitment to the product (Atakan et al., 2014).

We are aware about the main barrier of implementing these tools. It is related to the instrumental vision of Corporate Social Responsibility (Jensen, 2001), geared toward acquiring a brand image and obtaining a competitive advantage (Yelkikalan and Köse, 2012). Thus, the involvement of interested groups seems to be linked more to capital contribution than to achieving value through generating intangible good (Retolaza et al., 2014), ignoring a humanist approach aimed at people, their needs, and particularities (Melé, 2013).

That is why initiatives that try to show the commitment of the company with any social cause are frequently questioned. They are seen as manipulating actions of marketing, and the connection with and consistency of organizational actions are called into question (Ballabriga, 2009; Morata, 2010; Benavides, 2013).

In this context, these certificates could be misinterpreted; they could be understood that using them is not for the Common Good (Kuhn and Deetz, 2008; Zamagni, 2012; González-Fabre, 2015), but rather obeys other interests, whether it be the fulfillment of current regulations (Fernández, 2007; González, 2015), greater economic competitiveness (Friedman, 1970; Jensen, 2001), better brand positioning (Villagra and López, 2013; Benavides, 2015), or a method of obtaining social status (Yoon et al., 2006).

In any case, the existence of these kinds of certifications are making companies more ethical, trying to give a response to the concerns of others to make a better company for people with disabilities specifically and for society at large (Freeman, 1984).

## Conclusion

The main goal of this paper was to show that clear corporate information about the inclusion of people with disabilities might increase costumer loyalty.

As a result of our theoretical revision, we can say that this statement is consistent with the findings that we have exposed. In this sense, several arguments have been given to support it.

First of all, we have pointed out that disability is a phenomenon that concerns the entirety of human society, in the short or long term. Assuming this reality, the impairment that faces people with disabilities becomes an unacceptable social problem that companies should take into consideration if they want to be perceived as socially responsible.

Secondly, multiple researches show that companies that are considered as responsible are rewarded with the loyalty of the consumers. If those consumers are concerned about how the company is responding to the labor exclusion of people with disability, we have labeled them SRCs toward disability.

We have also shown that, in general terms, if the company is able to give clear information about how disability is treated, those responsible consumers will reinforce the loyalty toward the brand.

Even though we have underlined that the social exclusion is not really known by the society at large, companies are more and more aware about this question and they use different ways to show society their commitment to the problem. They may organize events to get direct acknowledgment from society because of their social actions in favor of people with disabilities, or they may implement programs to get an official certificate.

If we take into consideration that nowadays the collective is asking for being included in the companies and not for charitable actions, it is thinkable that they would consider that these certificates, CPDM as much as Bequal, more aligned to their own values than a social event that is hardly valuable.

Even though each certificate tends to evaluate different aspects the inclusion of disability in the company, both certificates together would reach the society as a whole. CPDM reminds that any disease can turn into disability and Bequal guarantees equal rights for all the citizens when going to work. Thus, any consumer properly informed about these questions may become a SRC toward disability and remain loyal to that brand or product. Even if we see this problem improbable to happen in our life, it can appear in any moment, and we all know it.

## Limitations and Future Research

Despite our enthusiasm and conviction that certifying the inclusion of people with disability can decisively contribute to improving the quality of life of people with disability. Therefore, to society at large, we are conscious that there is still a long way to go. Society has to become aware of the problem, and to accept that it can happen to anyone. So, awareness-raising education programs are needed, and they could be led by companies to change values among the citizens (Camacho et al., 2013).

We have also witnessed that there is little literature focused on disability and consumer loyalty, as we have pointed out in our revision. This may be due to the invisibility of the problem and the permanent exclusion of society that people with disability have been suffering for years. The lack of research in this area can mean that society is not interested in it. We suggest that it is a good time to change this situation through scientific studies.

Some questions are still pending from our theoretical revision:

• It would we important to compare the impact on loyalty after implementing each certificate.
• As the Bequal certificate is in its very beginning stage, it will be very interesting to see the obstacles that meet its implementation.

## Management Models Websites

• AA1000: www.accountability21.net/aa1000series
• ISO 26.000: www.iso.org/sr
• SA8000: www.sa8000.org
• SGE21: http://www.sge21.foretica.es/es/
• GRI : https://www.globalreporting.org/Pages/default.aspx

## Author Contributions

MG and JLF have worked together on the state of art of this review. They have also worked together on the design of the certification tool that we introduce in this article.

## Conflict of Interest Statement

The authors declare that the research was conducted in the absence of any commercial or financial relationships that could be construed as a potential conflict of interest.

## Footnotes

1 www.ieatraining.com

2 www.dmec.org

3 www.idmsc.org

4 http://www.bequal.es/sello_bequal.html.
